# Reimagining the Power of Nucleic Acids as Therapeutic and Diagnostic Agents

**DOI:** 10.3390/biom11111707

**Published:** 2021-11-17

**Authors:** Anthony Berdis

**Affiliations:** Department of Chemistry, Cleveland State University, 2351 Euclid Avenue, Cleveland, OH 44115, USA; a.berdis@csuohio.edu; Tel.: +1-216-867-2454

The central dogma of molecular biology proposes that in a typical cell, the flow of genetic information proceeds from DNA to RNA to polypeptide [1]. In this model, DNA is the genetic blueprint for an organism that must remain unchanged for the survival of an organism and for the propagation of a species. RNA then functions to decode the information present in the DNA blueprint, serving as the messenger needed to produce a specific protein that is the functional end-product of several processes encompassing transcription, RNA modification, and translation [2]. Over the past 60 years, this dogma has been revised to take into account seminal discoveries in nucleic acid function. For example, the discovery that RNA can be converted into DNA through the process of reverse transcription changed our understanding of viruses and had an incredible impact of molecular biology techniques [3]. Likewise, the discovery that RNA could, in some cases, catalyze reactions such as enzyme reactions was a paradigm shift in our understanding of molecular biology [4]. More recent discoveries, particularly in the area of RNA metabolism, have produced a deeper understanding of these processes, and in some instances, have generated new concepts that challenge this universality of the dogma of molecular biology. Information derived from these studies have led to the development of new methodologies to understand how gene expression affects cellular homeostasis and how perturbations in these processes can potentially lead to disease. This Special Issue contains five contributions from experts in the field and includes four reviews and one original research article encompassing diagnostics, therapeutics, synthetic biology, and other exciting areas in the chemistry and biology of DNA and RNA.

The research article by Hong et al. explores how RNA molecules can be applied in synthetic biological systems to create artificial platforms that can regulate complex cellular processes such as transcription and translation [5]. These authors utilized three specific types of de novo-designed synthetic RNA regulators to construct RNA-based synthetic gene circuits. These systems include small transcriptional-activating RNA (STAR), toehold switch (THS), and three-way junction (3WJ) repressor. The authors combined these regulators to first construct type 1 incoherent feed-forward loop (IFFL) circuits. They then explored their dynamic behaviors under in vivo conditions. The authors showed that these RNA-based regulators act much faster than the protein-based regulators that are typically found in cells. While the increased “speed” of the system suggests an improvement in signaling efficiency, the authors point out that the faster reaction kinetics obtained using RNA-based regulators may not always be beneficial, as they may adversely influence the functionality of a circuit. In addition, the authors demonstrate good agreement between their experimental data with theoretical simulations, suggesting that mechanistic modeling can be used in the future to validate the overall design of a new, synthetic circuit as well as to resolve potential experimental pitfalls. This body of research provides new directions in developing and optimizing new gene circuits that use RNA-based regulators.

The review article by Piotter, McClements, and MacLaren provides a comprehensive discussion on how nucleic acid technologies represent promising therapies against Stargardt disease, one of the most prevalent causes of inherited blindness in children [6]. While this disease can be identified at an early age, this condition unfortunately progresses over the lifetime of the afflicted individual. Despite early detection and slow rates in disease progression, there are no effective points for therapeutic interventions in most affected individuals. In their review article, the authors provide an excellent description of current therapeutic strategies such as small molecular weight compounds, anti-sense oligonucleotides, and cell replacement strategies and agents used to combat Stargardt disease. The authors describe a number of limitations to these approaches, leading to an excellent discussion on the potential utilization of CRISPR-based technologies to edit the genomes of afflicted patients as a way to combat Stargardt disease. The authors present recent pre-clinical and clinical trial data relating to the different strategies being applied to the problem of generating a treatment for the large cohort of Stargardt disease patients worldwide.

CRISPR–Cas systems are typically associated with gene-editing capabilities. However, there has been growing attention for applying CRISPR–Cas systems as diagnostic tools due to their ability to target specific genes. The review article by Kim, Ji, and Koh provides an excellent description of how CRISPR–Cas systems work [7]. Particular emphasis is placed on the protein components of the various systems in addition to the role and importance of the guide RNA that is needed to cleave target DNA or RNA. The authors describe applications of CRISPR–Cas systems in diagnostic testing methods that are based on target-specific binding. Their paper describes exciting applications in detecting disease-related genes, microRNAs, genetic variations such as single nucleotide polymorphism, and DNA methylation. Finally, the authors conclude their discussions by describing how CRISPR–Cas systems can be employed to detect non-nucleic acids targets such as proteins.

Flavonoids are a structurally diverse class of natural products that possess a number of beneficial activities in humans. Unfortunately, the widespread clinical use of these agents has been hampered due to a number of poor “drug-like” properties, including low solubility, chemical stability, bioavailability, and extensive in vivo metabolism. One strategy to overcome these limitations is to introduce site-specific modifications into flavonoids via selective methylation and/or glycosylation. An advantage of this approach is that these processes occur naturally in plants and thus could be used to improve their biophysical and pharmacokinetic properties. The review article by Sajid et al. [8] describes a new approach for efficient ex planta flavonoid synthesis using a combination of synthetic biology, metabolic engineering, protein engineering, and machine learning methods. Particular emphasis is placed on using synthetic biology methodologies to increase the methylation and glycosylation pathways, which can then produce modified flavonoids with improved pharmacodynamic and pharmacokinetic properties. Developing alternative production systems for the synthesis of flavonoids and their methylated and glycosylated forms will help facilitate their greater clinical application.

Even when present at low concentrations in water and soil, heavy metals such as mercury ion (Hg^2+^) are harmful to the health of humans and other animals. To help minimize the risk of accidental exposures, it is important to develop assays that can accurately measure the levels of poisonous metals such as Hg^2+^. While several methods have been developed using DNA biosensors, there are few reports integrating this technology with enzyme-driven signal amplification to improve the overall sensitivity for detecting and measuring Hg^2+^. The article by Wang provides an excellent review on emerging strategies in this innovative area [9]. The article provides an excellent description of the various enzymes such as nucleases and DNAzymes that are employed in this technique. A key innovation in this strategy relies on the ability of Hg^2+^ to convert single-stranded DNA into double stranded DNA through interactions with the nucleobase, thymine. The author also reviews how DNAzyme-assisted signal amplification strategies can be employed to detect several other metals, including Cu^2+^ and Mg^2+^.
